# Repair Strategy for Epoxy Anticorrosive Coatings on Steel Structures

**DOI:** 10.3390/ma16186257

**Published:** 2023-09-18

**Authors:** Kun Tu, Jin Wu, Xing Zhao, Lifang Zhang, Zehui Zhang

**Affiliations:** 1 Department of Civil and Airport Engineering, Nanjing University of Aeronautics and Astronautics, 29 Yudao Street, Nanjing 210016, China; tukun88811@126.com (K.T.);; 2 Jiangsu Airport Infrastructure Safety Engineering Research Center, 29 Yudao Street, Nanjing 210016, China

**Keywords:** steel structure, epoxy anticorrosive coating, repair strategy

## Abstract

This paper presents the results of a test of epoxy anticorrosion coating repair. Two strategies of coating repair were studied, respectively: partial repair and integral repair. The gloss loss rate, color difference and rust area rate of the repair coating were measured. The anticorrosion performance of repair coatings under different repair methods was compared. Based on the results of the coating repair test, the repair criteria for coatings were defined, and the repair strategy for epoxy anticorrosive coatings on steel structure was proposed. The results show that Marathon 500 super abrasion resistant epoxy paint (M500) and Jotamastic 90 GF low surface treatment epoxy abrasion resistant glass flake paint (J90GF) are suitable for partial repair of epoxy anticorrosive coatings on steel structures, and that Jotamastic 90 low surface treatment epoxy abrasion resistant coating (J90) and Marathon 500 super abrasion resistant epoxy paint (M500) are suitable for integral repair of epoxy anticorrosive coatings on steel structures.

## 1. Introduction

Steel structures are corroded by environmental factors such as water and oxygen [1]. Coating anticorrosion material on the surface of a steel structure is the most economical, effective and widely used anticorrosion means [2,3,4]. However, the anticorrosion coating of a steel structure will also be affected by various factors, such as ultraviolet light, temperature, water, oxygen, load, etc., which will lead to the exposure, light loss, discoloration, pulverization, blistering, rust, and peeling of the anticorrosion coating [5,6,7,8].

The target life of a steel structure is much longer than that of its anticorrosion coating. For example, the life of a bridge is generally 100 years, while the life of an anticorrosion coating is less than 30 years [9,10,11]. Thus, the repair and maintenance of the anticorrosion coating is an important factor in the service of structures. The coatings subjected to external forces or that have reached the end of their service life (corrosion, blistering, peeling, and so on) all indicate that coatings need to be repaired. The annual cost of steel structure maintenance worldwide is huge. However, people pay little attention to coating maintenance. Andreas et al. [12] studied the effects of surface treatment and coating type on the anticorrosion performance of offshore steel structures. The three surface treatment methods were sand blasting, mechanical grinding and impact treatment. According to ISO 20340, an accelerated cycle exposure test was carried out on the repaired coating system. The results showed that the combination of dry blast cleaning and lower coating thickness provided the best protection performance. A higher coating thickness was recommended to maximize the anticorrosion performance in case blast cleaning was not feasible. Nicolaia et al. [13] explored measures to minimize the expected coating maintenance cost in finite time and infinite time. The results showed that the optimal maintenance scheme was the solution of a continuous time renewal dynamic programming equation. Coating maintenance is the main part of steel structure maintenance. However, studies on the process, construction technology and design theory of maintenance coatings are still scarce compared to those on new coatings [14].

On the premise of ensuring the stability and safety of a steel structure, significant savings in maintenance costs can be achieved if coatings with serious corrosion are repaired and coatings with good protective performance are retained [15]. The corrosion of a steel structure starts from the corrosion failure of the anticorrosion coating. Epoxy anticorrosion coatings are the most widely used anticorrosion paints. Therefore, research on repair strategies for epoxy anticorrosion coatings of steel structures have very important theoretical significance and engineering application value. In this study, a coating repair test was carried out on an epoxy anticorrosion coating, and two strategies of coating repair were studied, respectively: partial repair and integral repair. Changes in the light loss rate, color differences and rust area rate of the repair coating were mainly measured, and the anticorrosion performance of the repair coating under the partial repair and integral repair method was compared. Based on the results of coating repair tests, the repair criteria for the coatings were defined, and a repair strategy for epoxy anticorrosion coatings on steel structures was put forward.

## 2. Experimental Programs

### 2.1. Materials

The type of steel used in this study was Q235. The elastic modulus of the steel was 200 GPa, and the size of the specimen was 140 mm × 70 mm × 4.5 mm. The yield strength of the steel was 325 MPa, the tensile strength was 492 MPa, and the elongation was 36%. Before coating, specimens were mechanically polished to St2, and then degreased with metal cleaning agent and dehydrated with absolute ethanol according to China standard GB/T 8923-2011 [16]. The specimens were placed in a drying oven after being air dried.

The coatings were provided by Jotun coatings Co., Ltd. in Zhangjiagang, Jiangsu, China. Jotamastic 90 GF low surface treatment epoxy wear-resistant glass flake paint (J90GF) and Marathon 500 super wear-resistant epoxy paint (M500) were selected for partial repair, because Marathon 500 and J90GF are widely used in steel structures in China. The chemical composition of M500 included epoxy resin, anhydrides, and volatile solvents. The chemical composition of J90GF included epoxy resin amines, toluene, silicon dioxide (SiO_2_), sodium oxide (Na_2_O) and potassium oxide (K_2_O). Jotamastic 90 low surface treatment epoxy wear-resistant coating (J90), Jotamastic 90 GF low surface treatment epoxy wear-resistant glass flake paint (J90GF) and Marathon 500 super wear-resistant epoxy paint (M500) were used for integral repair. According to the Chinese code “Paints and varnishes—Determination of resistance to cyclic corrosion condition”, the results of the salt fog test indicated that the duration of resistance to corrosion for Marathon 500 (M500) and Jotamastic 90 GF (J90GF) coatings were 4000 h and 2000 h, respectively. The details of the coating systems are shown in Table 1. The airless spraying method was used for spraying. The coating operation was carried out in a well-ventilated room, and the coatings were coated on the surface of the specimens evenly. The specimens were cured at 25 °C for 24 h after coating.

### 2.2. Test Specimens for Repair Coating

Thirty-six specimens were prepared for repair coating, including 12 partial repair coating specimens and 24 integral repair coating specimens. The 12 specimens for the partial repair coating test were placed in different corrosive environments, and the specimen numbers are shown in Table 2.

Tensile stresses of 0 MPa, 65 MPa, 130 MPa and 195 MPa were applied to the specimens for the integral repair coating test, and specimens were placed in different corrosive environments (fresh water environment and saline environment). The specimen numbers are shown in Table 3.

**Figure 1:**
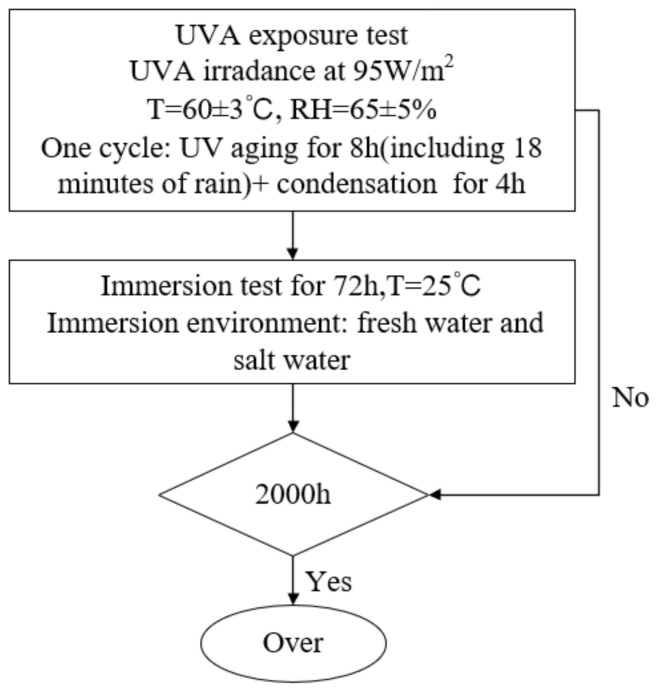
The procedure for the accelerated exposure test.

The accelerated exposure test was used in this study. Based on the service environment of the epoxy anticorrosion coating, the accelerated exposure test procedure shown in Figure 1 was designed. Specimens were placed in a fluorescent UV-condensation test chamber and then immersed in water tanks. Solutions in the two water tanks were fresh water and salt water (3.5% NaCl solution), respectively. The solutions in the water tanks were replaced once a week. Accelerated ageing tests were performed for a total of 2000 h. According to the standard of China (GB/T 9754-2007) [17], the gloss values of the organic coatings were measured with a 60° incident angle specular gloss tester (3NH-60) manufactured by Shenzhen 3NH Technology Co., Ltd. (Shenzhen, China). Three points were measured for each specimen. Coating thickness was measured by a CT-220 magnetic coating.

#### 2.2.1. Details of the Methods for Partial Repair of Coatings

**Figure 2:**
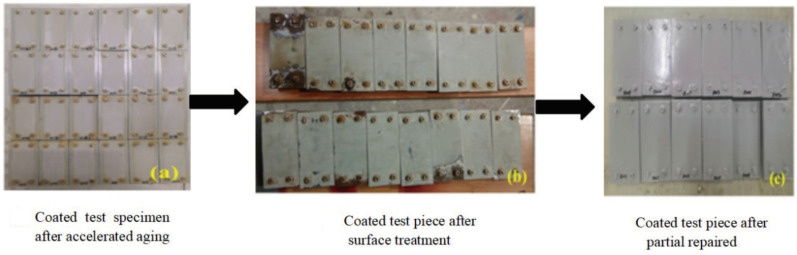
Preparation process for partially repaired coating test specimens.

**Figure 3:**
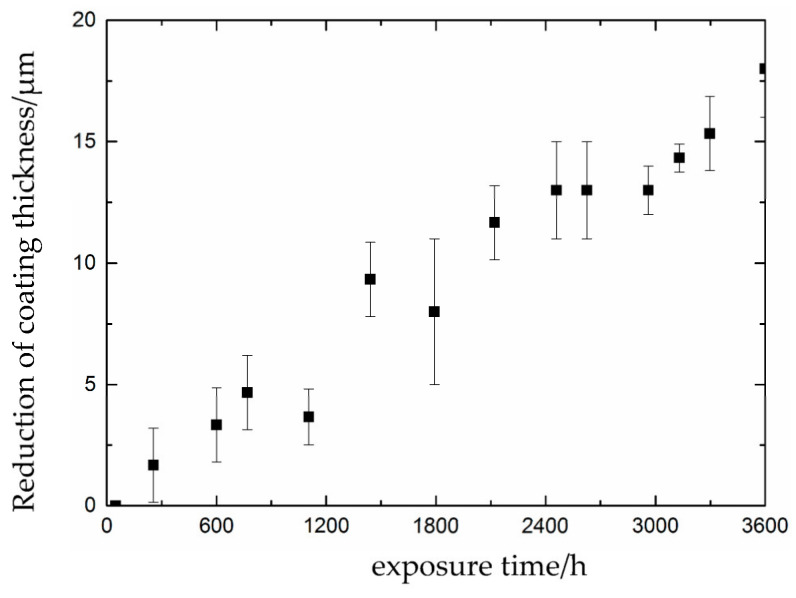
Reduction in coating thickness versus exposure time.

Figure 2 shows the preparation process for partially repaired coating specimens. Twelve of 24 specimens that underwent a 2000 h accelerated exposure test were used for the partial repair test. Figure 2a shows the accelerated exposure coating specimens. After 3600 h of the UV accelerated exposure test, the J90 coating lost light and was seriously discolored, and the coating thickness had decreased, as shown in Figure 3. It was found that the reduction in coating thickness increased significantly with exposure time, and the reduction in coating thickness reached 18 µm. Additionally, the coating had many corrosion points but still had a certain protective effect on the metal substrate. Therefore, partial repair was carried out on the J90 coating.

First, the old coating finishes were removed, then the coatings in the severely rusted zone were cleaned to the undamaged zone, and the grooves were processed in the old coating to bond the new coating well. The surface treatment grade was St3. The coated test specimens after surface treatment are shown in Figure 3b. Then, M500 and J90GF coatings were applied to the severely rusted zones of the specimens, respectively, and the accelerated exposure test and tensile loading were applied to, the partially repaired coating specimens for 2000 h. Partially repaired coating specimens are shown in Figure 3c.

#### 2.2.2. Details of the Methods for Integral Repair of Coatings

**Figure 4:**
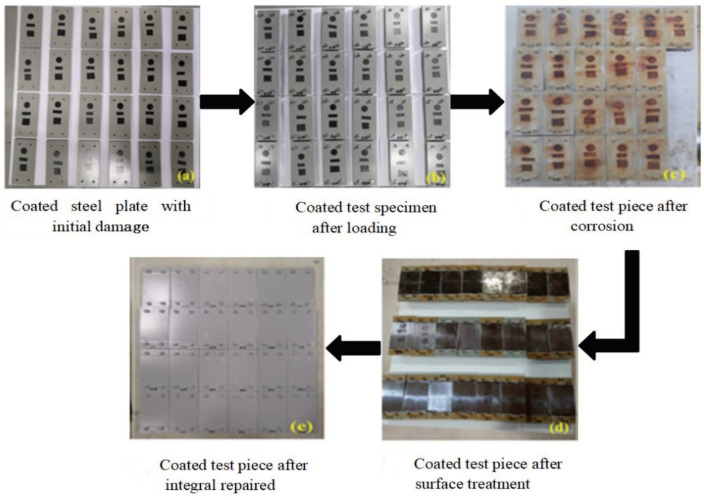
Preparation process for integrally repaired coating test specimens.

Figure 4 shows the preparation process for specimens used in the integral repair coating test. Figure 4a shows a coated specimen with initial damage. In order to shorten the test cycle, artificial initial damage was applied to the coating surface of the specimen. After the surfaces of the steel specimens were polished, small areas of the surfaces of the steel specimens were sealed with adhesive tape. Then, 24 coated specimens with initial damage and 24 loaded specimens were assembled as a whole, and a load was applied to obtain the loaded test specimens shown in Figure 4b. After a loaded coating specimen was sealed with epoxy resin, it was placed in an accelerated exposure environment for 2000 h under artificially accelerated corrosion. Figure 4c shows the corroded coating specimens. Surface treatment was applied to the corroded test specimens; the old paint layer was removed to the bolt anchorage zone, the bare metal matrix was mechanically polished, and the rust was removed. The coated test specimens after surface treatment are shown in Figure 4d. The integral repair method was adopted for the coated test specimens after surface treatment. The coating method was airless spraying, and the coatings selected for integral repair were J90, J90GF and M500. The coated test specimens are shown in Figure 4c. Then, the specimens were placed in an accelerated corrosion environment for 2000 h.

### 2.3. Performance Testing

A specular gloss meter was used to measure the gloss change of the coating; the instrument needed to be calibrated on a standard mirror before measurement. Five points were measured for each test piece. The average value was calculated to obtain the gloss of the coated test specimen, and finally the gloss loss rate of the coating was obtained according to GB/T 1766-2008 [18]. A Ct-220 coating thickness gauge was used to measure the change in thickness of the coating during exposure. Before measurement, the instrument was calibrated on a standard metal block to confirm that the measurement accuracy met the requirements. Five points were measured for each test piece, and then the average value was calculated to obtain the coating thickness of the coated test specimens. A camera was used to collect photos of the coating surface, and the color difference change of the coated specimen was calculated using the MATLAB 2014 Ra color difference program. The change in the rust area rate of the J90 coating was calculated by visual observation with a magnifying glass and image processing, and an optical microscopic image of the coating was with a Hirox three-dimensional digital microscope.

## 3. Results and Discussion

### 3.1. Partial Repair Test Results for Epoxy Anti-Corrosion Coating

**Figure 5:**
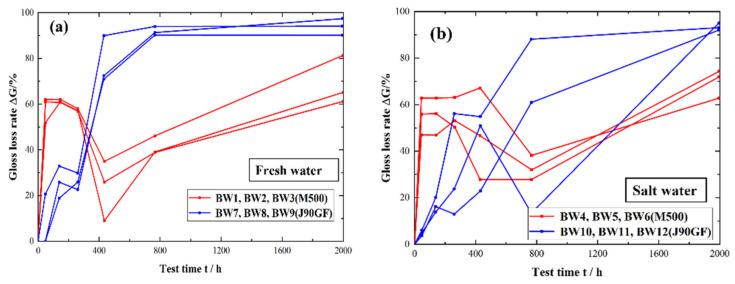
Light loss rate for two types of partially repaired coatings. (**a**) Fresh water; (**b**) saline water.

Figure 5 is a comparison of the change in the light loss rate for two types of partially repaired coatings. The change in the light loss rate for the two types of coatings was generally similar. In the first stage, the light loss rate increased rapidly. In the second stage, it decreased. In the third stage, the light loss rate increased again. The decrease in the light loss rate, referred to as the gloss recovery of the coating, was due to the temperature difference in the fluorescent UV-condensation test chamber and immersion in the water tanks. It can be seen from Figure 5a that there are still some variations in the light loss rate for the two types of partially repaired coatings in the fresh water. In the first stage of exposure (0 h–400 h), the light loss rate for the two types of partially repaired coatings increased for M500 compared to J90GF, which showed that the M500 coating was more prone to light loss, that is, the light retention of the two partial maintenance coatings was greater for J90GF than M500. It can be seen from Figure 5b that the change in the light loss rate for the two types of partially repaired coatings in the saline environment was similar to that in fresh water. But the gloss recovery in the saline environment was slower than that in the fresh water because the gloss recovery of coating was restrained by chloride ions in the saline environment [19].

**Figure 6:**
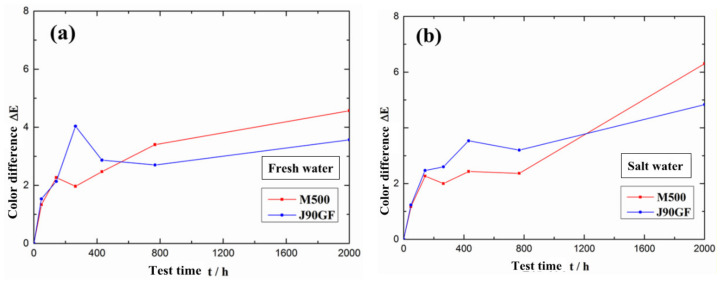
Average color difference of two types of partially repaired coatings. (**a**) Fresh water; (**b**) saline water.

Figure 6 is a comparison of the variations in the average color difference of the two types of partially repaired coatings. In the early stage of exposure, the average color difference of the J90GF coating was greater than that of the M500 coating, but after exposure for 2000 h, the average color difference of the M500 coating was greater than that of the J90GF coating. Thus, the discoloration of the J90GF coating was more pronounced in the early stage of exposure. Through the analysis of color difference, it was found that in the early stage of exposure, the coating discoloration was more severe for J90GF than M500. After exposure for 2000 h, the average color difference of the coatings in saline water was significantly larger than that in fresh water because of the action of chloride ions in saline water.

With the increase in exposure time, the corrosion of the coating gradually intensified. There were not only loss of light, discoloration, pulverization and other phenomena on the coating, but also serious defects such as rust. The rust on the coating was visually observed with a 10× magnifying glass. It was found that after 2000 h of comprehensive accelerated exposure, some repaired coating specimens showed lost light, discoloration and pulverization, but there were no serious defects such as rust. This was because the corroded coating specimens had been partially repaired. The corrosion of these corroded coating specimens was not particularly serious, but the finish paint had slight exposure defects such as loss of light, discoloration and pulverization, a reduced coating thickness, and many corrosion points on the coating, but the bottom coating was still able to provide a certain degree of protection to the metal matrix. In addition, for the partial repair, only the topcoat had been removed, while the bottom coating with a thickness of about 150 µm had been retained, and a low surface treatment repair coating with a thickness of about 290 µm had been applied, such that the total thickness of the coating on the specimen originally to be repaired reached 440 µm. Thus, the coating on the specimen after partial repair had better anticorrosion performance than the original one.

**Figure 7:**
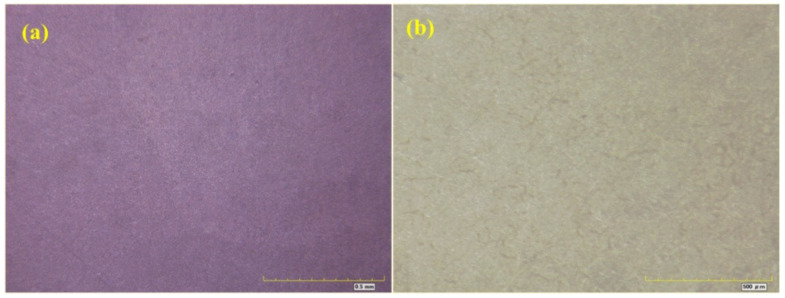
Optical micrograph of BW1. (**a**) 336 h; (**b**) 2000 h.

**Figure 8:**
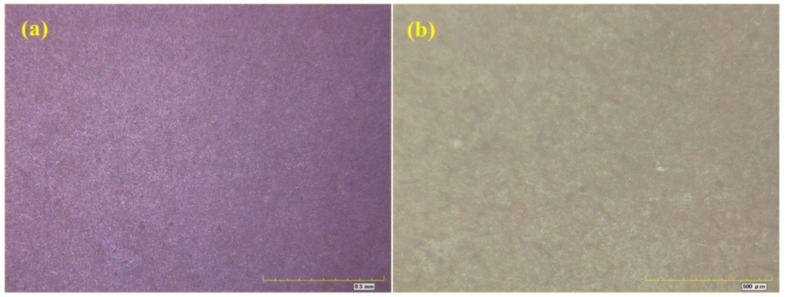
Optical micrograph of BW4. (**a**) 336 h; (**b**) 2000 h.

**Figure 9:**
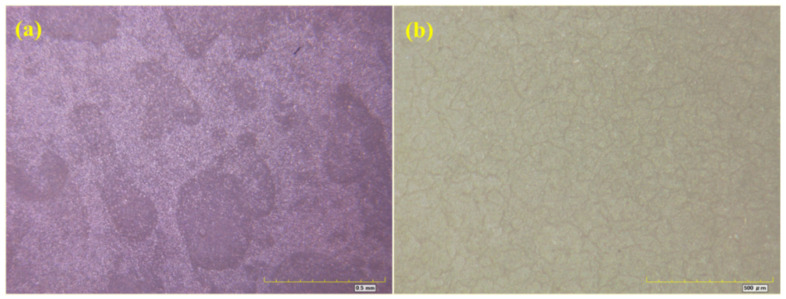
Optical micrograph of BW7. (**a**) 336 h; (**b**) 2000 h.

**Figure 10:**
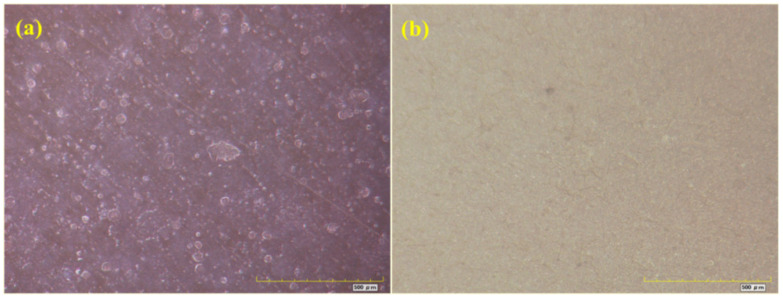
Optical micrograph of BW10. (**a**) 336 h; (**b**) 2000 h.

Figure 7, Figure 8, Figure 9 and Figure 10 show optical micrographs of the coating morphology of the coated specimens BW1, BW4, BW7 and BW10 after exposure for 336 h and 2000 h, respectively. BW1 was a specimen with an M500 coating in the fresh water environment. It can be seen from the 200 magnification optical micrograph in Figure 7 that BW1 did not change significantly after exposure for 336 h, i.e., 2 weeks, whereas after exposure for 2000 h, the color of the coating changed significantly and a few micro cracks appeared on the surface. BW4 was a specimen with an M500 coating in the saline environment. It can be seen from the 200 magnification optical micrograph in Figure 8 that the changes in the coating appearance of BW1 and BW4 were similar. BW7 was a specimen with a J90GF coating in the fresh water environment. With the increase in exposure time, loss of light, discoloration and pulverization gradually appeared on BW7. It can be seen from the 200 magnification optical micrograph in Figure 9 that the coating appearance of BW7 changed slightly after exposure for 336 h, i.e., 2 weeks, and there were clearly defined irregular spots on the coating, whereas after exposure for 2000 h, the color of the coating changed significantly, and many micro cracks appeared on the surface. BW10 was a specimen with a J90GF coating in the saline environment. It can be seen from the 200 magnification optical micrograph in Figure 10 that BW10 changed slightly after exposure for 336 h, i.e., 2 weeks, and there were many spots on the coating. However, after exposure for 2000 h, the color of the coating changed significantly, and a few micro cracks appeared on the surface. Comparing BW1 with BW7, it can be seen from the optical micrographs that there were more spots and micro cracks on BW7, which indicates that the corrosion of the J90GF coating was more serious in the fresh water environment. Comparing BW4 with BW10, it was found that the corrosion of the J90GF coating was more serious in the saline environment because of the action of chloride ions. Thus, from the appearance of the coating, the coating corrosion was more severe for J90GF than for M500.

In general, the trends of change in the light loss rate, color difference and corrosion of the coatings were similar in the fresh water and saline water environments, but the light loss rate, color difference and corrosion of the coatings with J90GF were larger than those of the coatings with M500. The corrosion of coatings in the saline environment was more serious because of the action of chloride ions.

### 3.2. Integral Repair Test Results for Epoxy Anti-Corrosion Coating

**Figure 11:**
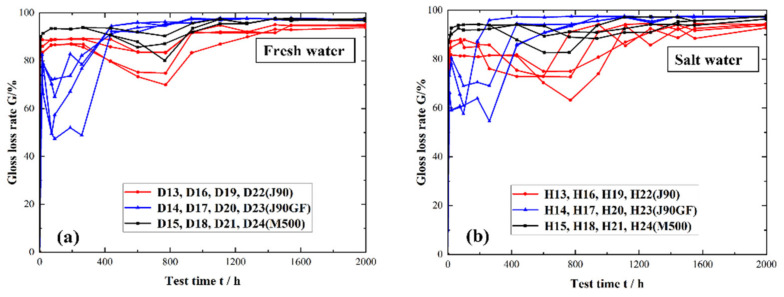
Light loss rates of three types of integral repair coatings. (**a**) Freshwater environment; (**b**) saline environment.

Figure 11 shows a comparison of the change in the light loss rate of three integral repair coatings. The change in the light loss rate of the three integral repair coatings was generally similar. In the early stage, the light loss rate increased rapidly, and then the growth rate slowed down. Finally, the change in the light loss rate tended to stabilize, and the coatings lost light completely. It can be seen from Figure 11a that there were still some differences in the light loss rates of the three integral repair coatings in the fresh water environment. In the early stage of exposure (0 h–400 h), the rate of increase in light loss of the three maintenance coatings was faster for M500, followed by J90 and then J90GF, which shows that the M500 coating was the most prone to light loss, and the J90GF coating was the least prone, that is, the light retention of the three integral repair coatings was highest for J90GF, followed by J90 and then M500. As shown in Figure 11, the gloss recovery of the three integral repair coatings could be explained by the fact that the temperature of the coating specimen in the ultraviolet exposure box was about 60 °C, and the temperature during the immersion test was ambient or room temperature. The gloss would increase in a short time and the light loss rate would decrease in a short time if the temperature of the coating rose; this phenomenon is called gloss recovery. Scrinzi et al. [19] discovered a similar phenomenon, namely that the temperature difference may lead to gloss recovery, and confirmed the effect of temperature on gloss recovery through heat treatment. The influence of temperature difference on the three integral repair coatings was strongest for J90GF, followed by J90 and then M500. It can be seen from Figure 11b that the change in the light loss rate of the three integral repair coatings in the saline environment was similar to that in fresh water. The gloss recovery phenomenon is conducive to maintaining the gloss of the coating, so the more obvious the gloss recovery phenomenon is, the more conducive it is to the gloss retention of the coating. From the research on the light loss rate, it was found that the light retention performance of the three integral repair coatings was best for J90GF, followed by J90 and then M500. The gloss recovery of the integral repair coatings in the saline environment was smaller than that in fresh water, it is because of the action of chloride ions in saline environment.

**Figure 12:**
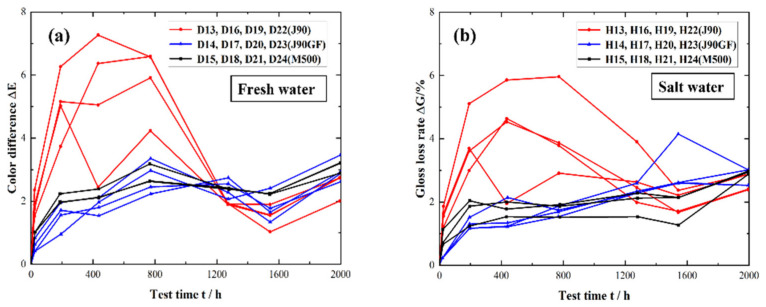
Color difference of three types of integral repair coatings. (**a**) Freshwater environment; (**b**) saline environment.

Figure 12 shows the color difference changes in the three integral repair coatings. Figure 12a shows the color difference changes in the three integral repair coatings in the freshwater environment, and Figure 12b shows the color difference changes in the three integral repair coatings in the saline water environment. As can be seen from Figure 12a, in the early stage of exposure, the color difference of the J90GF coating was greater than that of the J90 coating and M500 coating, while the color differences of the J90 and M500 coatings were slight compared with that of J90GF. As can be seen from Figure 12b, the color differences of the three integral repair coatings in the saline environment were similar to those in the freshwater environment. Thus, it was found that in the early stage of exposure, the coating discoloration was most severe with the J90GF coating, followed by J90 and then M500.

**Figure 13:**
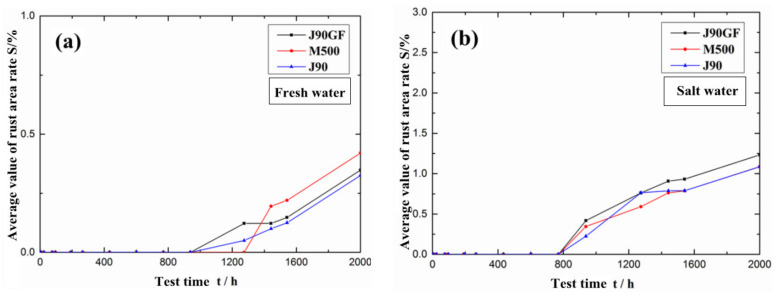
Rust area rate of three types of integral repair coatings. (**a**) Freshwater environment; (**b**) saline environment.

Figure 13 shows the rate of change in the rust area of the three integral repair coatings. It can be seen from Figure 13a that in the freshwater environment, the corrosion of the M500 coating was obviously more serious than that of the J90 coating and J90GF coating. After the 2000 h comprehensive accelerated exposure test of the three repair coating systems, the corrosion severity in descending order, as measured in rust area, was M500 > J90GF > J90. It can be seen from Figure 13b that the J90GF coating had more serious corrosion than the M500 and J90 coatings in the saline environment. After the 2000 h comprehensive accelerated exposure test, in terms of rust area, the corrosion severity, in descending order, of the three repair coating systems was J90GF > M500≈J90. After exposure for 2000 h, the coatings in the saline environment showed more serious corrosion than in the freshwater environment. This is because the saline corrosion was more extensive than the freshwater corrosion.

**Figure 14:**
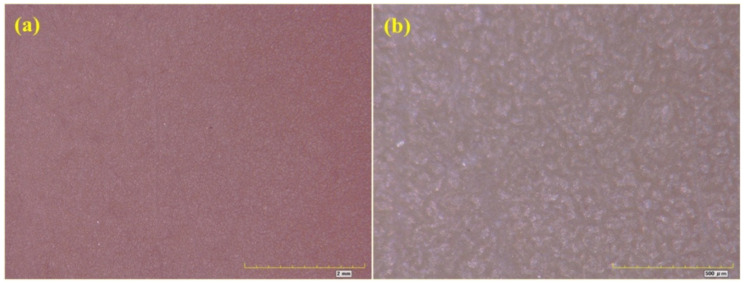
Optical micrograph of D15. (**a**) 50; (**b**) 200.

**Figure 15:**
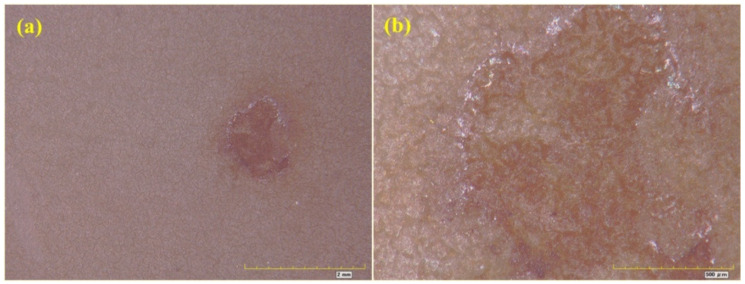
Optical micrograph of H15. (**a**) 50; (**b**) 200.

From the exposure rating shown in Table 4, it was found that D15 showed loss of light, discoloration and pulverization in the early stage, but there were a few rust spots on the coating at about 1106 h, and the rust grade was Ri1. After exposure for 2000 h, although the rust area of the coating had expanded, the rust grade was still Ri1. Figure 14 shows an optical micrograph of coated specimen D15 after exposure for 2000 h. The magnification of the optical images shown in Figure 14a,b was 50 times and 200 times, respectively. There were not many rust spots on the coated specimen D15 after exposure for 2000 h, but there were some micro cracks on the coating. Generally speaking, the corrosion of specimen D15 was slight. From the exposure rating shown in Table 4, it was found that H15 showed loss of light, discoloration and pulverization in the early stage, but a small number of rust spots and bubbles appeared on the coating at about 1106 h; the rust grade was Ri2, and the foaming grade was 1(S1). After exposure for 2000 h, the extent of pulverization was more serious, the rust area of the coating had gradually expanded, and the rust grade was Ri3. Figure 15 shows an optical micrograph of H15 after exposure for 2000 h. The magnification of the optical images shown in Figure 15a,b was 50 and 200 times, respectively. It can be seen from Figure 15 that H15 had been seriously corroded after exposure for 2000 h.

In general, the light loss rate of the coatings with M500 was larger than that with J90GF and J90, the color difference of the coatings with J90GF was larger than that with J90 and M500, and the corrosion of the coatings with J90GF was larger than that with M500 and J90. The corrosion of the coatings in the saline environment was more serious than that in the freshwater environment.

### 3.3. Repair Strategy for Epoxy Anticorrosive Coatings on Steel Structures

Based on the analysis of the repair test results in this study, the repair strategy for epoxy anticorrosion coatings on steel structures was explored. For the partial repair test, the selected repair coatings were the J90GF coating and M500 coating. These two coatings were low surface treatment coatings, which are suitable for coating repair. From the standpoint of light loss rate, the anticorrosion effect of the J90GF coating was better than that of the M500 coating. From the standpoint of color difference, the anticorrosion effect of the M500 coating was better than that of the J90GF coating. From the standpoint of rust area, after the 2000 h comprehensive accelerated exposure test, there was no rust on the two partial repair coatings, but no further accelerated exposure testing was carried out in this study. Therefore, strictly speaking, under the accelerated exposure environment, after 2000 h comprehensive accelerated exposure, the anticorrosion effects of the J90GF and M500 coatings were similar. The light loss rate and color difference are indexes used to evaluate the early exposure conditions of coatings. From these two indexes, the J90GF coating and M500 coating had their own advantages and disadvantages. The rust area rate is an index to evaluate the exposure condition of a coating in the middle and late stages. From this index, there was no significant difference between the J90GF coating and M500 coating. In conclusion, both maintenance coatings are suitable for partial maintenance of anti-corrosion coatings.

For the integral repair test, the selected maintenance coatings were the J90 coating, J90GF coating and M500 coating. From the standpoint of light loss rate, the anticorrosion effects of the three coatings were J90GF > J90 > M500, from better to worse. From the standpoint of color difference, the anticorrosion effects of the three coatings were J90 ≈ M500 > J90GF. From the standpoint of rust area, after the 2000 h comprehensive accelerated exposure test, the anticorrosion effects of the three coatings were M500 ≈ J90 > J90GF. In conclusion, although the three coatings are low surface treatment coatings, the J90GF coating had the most serious corrosion after the 2000 h comprehensive accelerated exposure test. Therefore, the J90 coating and M500 coating are more suitable for the integral repair of anticorrosion coatings.

It is worth discussing how to define the repair criteria (when the coating needs to be repaired) for an anticorrosion coating on a steel structure. Referring to the technical requirements for repair coating and recoating in JT/T 722, combined with the test results of this study, the criteria for partial repair are defined as follows: (1) the finish paint has pulverization of grade 3 or more, and the thickness after pulverization thinning is greater than 50% of the initial thickness; (2) the coating is in grade 2~3 cracking or grade 2~3 peeling or grade 2~3 blistering, but the primer is intact; (3) the rust area rate of the coating is 0.5~1%. The criteria for integral repair are defined as follows: (1) the rust area rate of the coating reaches more than 5%; (2) the coating is cracked above grade 3 or peeled off above grade 3 or blistered above grade 3.

The repair strategy for epoxy anti-corrosion coatings on steel structures is listed in Table 5. The recommended repair coatings in Table 5 are based on the conclusions of the repair tests in this study. The application of other repair coatings needs further research.

## 4. Conclusions

(1) From the standpoints of light loss rate, color difference and rust area rate, it was found that Marathon 500 super wear-resistant epoxy paint and Jotamastic 90 GF low surface treatment epoxy wear-resistant glass flake paint are suitable for the partial repair of epoxy anti-corrosion coatings on steel structures.
(2) From the standpoints of light loss rate, color difference and rust area rate, it was found that Jotamastic 90 low surface treatment epoxy wear-resistant coating and Marathon 500 super wear-resistant epoxy paint are more suitable for the integral repair of epoxy anticorrosion coatings on steel structures.
(3) Based on the results of coating repair tests, the repair criteria for partial and integral repairs were defined, respectively, and the repair strategy for epoxy anticorrosion coatings on steel structures was put forward.

## Figures and Tables

**Table 1:** Details of the Coating Systems.

Parameters	Coating Systems		
	J90	J90GF	M500
Volume solid content	80 ± 2%	80 ± 2%	85 ± 2%
Gloss (GU 60°)	Semi-gloss (35–70)	Semi-gloss (35–70)	Gloss (70–85)
Flash point	35 °C	35 °C	43 °C
Density	1.4 kg/L	1.4 kg/L	1.6 kg/L
VOC	246 g/L	224 g/L	162 g/L
Color	Grey	Grey	Grey
Mixing ratio (volume)	A:B = 3.5:1	A:B = 3.5:1	A:B = 5:1
Diluent	Jotun 17# diluent	Jotun 17# diluent	Jotun 17# diluent

**Table 2:** Specimen Numbers for Partial Repair Test.

Test	Coating System	Coating Thickness (µm)	Surface Treatment Grade	Corrosive Environment	Number	Label
Partial repair test	M500	290 ± 20 (Bottom integration)	St3	Fresh water	3	BW1, BW2, BW3
				Saline water	3	BW4, BW5, BW6
	J90GF			Fresh water	3	BW7, BW8, BW9
				Saline water	3	BW10, BW11, BW12

**Table 3:** Specimen Numbers for Integral Repair Test.

Test	Coating System	Coating Thickness (µm)	Surface Treatment Grade	Corrosive Environment	Number	Label
Integral repair test	J90GF	290 ± 20 (Bottom integration)	St3	Fresh water	4	D13, D16, D19, D22
				Saline water	4	H13, H16, H19, H22
	M500			Fresh water	4	D14, D17, D20, D23
				Saline water	4	H14, H17, H20, H23
	J90			Fresh water	4	D15, D18, D21, D24
				Saline water	4	H15, H18, H21, H24

**Table 4:** Exposure Rating of Integrally Repaired Coating Specimens.

Item	Exposure/h	Pulverization	Cracking	Blistering	Rust	Spalling	Comprehensive Rating
D15	434	0	0	0	Ri0	0	0
	1106	1	0	0	Ri1	0	1
	2000	2	0	0	Ri1	0	2
H15	434	0	0	0	Ri0	0	0
	1106	2	0	1(S1)	Ri2	0	2
	2000	3	0	1(S1)	Ri3	0	4

**Table 5:** Repair Strategy for Epoxy Anti-corrosion Coatings on Steel Structures.

Repair Strategy	Repair Criteria	Repair Coating	Repair Method
Partial repair	(1) The finish paint has pulverization of grade 3 or more, and the thickness after pulverization thinning is greater than 50% of the initial thickness; (2) The coating is in grade 2~3 cracking or grade 2~3 peeling or grade 2~3 blistering, but the primer is intact; (3) The rust area rate of the coating is 0.5%~1%.	M500, J90GF	Remove the finish coating and clean the coating in the severely rusted area to the undamaged area, groove the damaged and undamaged area joints, treat the surface to Sa2 or St3, and apply a certain thickness of low surface treatment repair coating.
Integral repair	(1) The rust area rate of the coating reaches more than 5%; (2) The coating is cracked above grade 3 or peeled off above grade 3 or blistered above grade 3.	J90, M500	Conduct thorough surface treatment on the corroded coating, remove the old paint layer to the bolt anchorage area, mechanically polish the exposed metal matrix, and apply a certain thickness of low surface treatment repair coating on the surface of the treated steel structure.

## Data Availability

Not applicable.
